# The rich inner life of the cell nucleus: dynamic organization, active flows, and emergent rheology

**DOI:** 10.1007/s12551-020-00761-x

**Published:** 2020-10-16

**Authors:** Alexandra Zidovska

**Affiliations:** Center for Soft Matter Research, Department of Physics, New York University, New York, NY USA

**Keywords:** Cell nucleus, Chromatin dynamics, Nuclear compartmentalization, Active matter, Rheology

## Abstract

The cell nucleus stores the genetic material essential for life, and provides the environment for transcription, maintenance, and replication of the genome. Moreover, the nucleoplasm is filled with subnuclear bodies such as nucleoli that are responsible for other vital functions. Overall, the nucleus presents a highly heterogeneous and dynamic environment with diverse functionality. Here, we propose that its biophysical complexity can be organized around three inter-related and interactive facets: heterogeneity, activity, and rheology. Most nuclear constituents are sites of active, ATP-dependent processes and are thus inherently dynamic: The genome undergoes constant rearrangement, the nuclear envelope flickers and fluctuates, nucleoli migrate and coalesce, and many of these events are mediated by nucleoplasmic flows and interactions. And yet there is spatiotemporal organization in terms of hierarchical structure of the genome, its coherently moving regions and membrane-less compartmentalization via phase-separated nucleoplasmic constituents. Moreover, the non-equilibrium or activity-driven nature of the nucleus gives rise to emergent rheology and material properties that impact all cellular processes via the central dogma of molecular biology. New biophysical insights into the cell nucleus can come from appreciating this rich inner life.

## Introduction

The cell nucleus is arguably one of the most important organelles in eukaryotic cell, housing the genome that contains the genetic blueprint for the entire cell (Alberts et al. 2014). The genetic information is stored in the DNA molecule, which lies at the core of the central dogma of molecular biology (Crick 1958, 1970). DNA is transcribed into RNA, which becomes translated into proteins. The first step of gene expression, transcription, occurs in the cell nucleus assisted by the intricate interplay of molecular machinery that acts on chromatin, the functional form of DNA inside cells (Van Holde 2012; Alberts et al. 2014). In addition, many other DNA transactions occur inside the nucleus such as genome replication prior to cell division or DNA repair to maintain genome integrity. These processes are ATP-dependent and their molecular machinery requires direct access to the DNA molecule, leading to a persistent dynamic rearrangement of the genome. While the biochemistry of these processes has been studied in great detail (Van Holde 2012; Alberts et al. 2014), their biophysical mechanisms and implications are far from understood (Hübner and Spector 2010; Dekker et al. 2013; Gibcus and Dekker 2013; Bickmore and van Steensel 2013; Sazer and Schiessel 2018). Moreover, the timescales and length scales of these processes are directly influenced by the material properties of the nucleus and its constituents, which in turn affect all cellular processes via the central dogma. For example, the viscosity of the nucleoplasm impacts the rates of molecular and organelle transport inside the nucleus, whereas the persistence length of the DNA molecule affects its local organization and dynamics (Milo and Phillips 2015).

In addition to the highly dynamic genome, the nucleus contains a plethora of smaller structures such as nucleoli, Cajal bodies, PML bodies, and speckles (Misteli and Spector 2011; Alberts et al. 2014). These subnuclear bodies serve as sites of further essential processes and often migrate and undergo their own dynamic rearrangement or restructuring, e.g., the coalescence of nucleoli or speckles (Caragine et al. 2018; 2019; Kim et al. 2019). The genome and subnuclear bodies are all immersed in the nucleoplasm, a surrounding fluid rich with proteinaceous molecular machinery as well as their respective molecular products such as RNA. This complex solution of polymers and colloidal particles is confined by the nuclear envelope that is comprised of a layer of intermediate filaments called lamins and two lipid bilayers (Alberts et al. 2014). Very recently, the nuclear envelope was found to be perpetually undulating (Chu et al. 2017).

Overall, the cell nucleus is a rich environment with a rich inner life. Its constituents are numerous and diverse, ranging from polymers to colloids, from small molecules to macromolecules, giving rise to a highly heterogeneous system. Strikingly, the nucleus lacks any internal boundaries, yet its content is evidently functionally organized. Moreover, its organization is dynamical, simultaneously accommodating many orthogonal active (ATP-dependent) processes happening concurrently, and thus giving rise to emergent behaviors and properties. Hence, the cell nucleus presents a non-equilibrium living system, which defies principles of equilibrium thermodynamics. In this review, we will survey current knowledge about the biophysical origins of nuclear organization and heterogeneity, dynamics of nuclear constituents, nuclear compartmentalization via phase separations, and the emergent rheology of the nucleus.

## Nuclear organization and heterogeneity

**Fig. 1:**
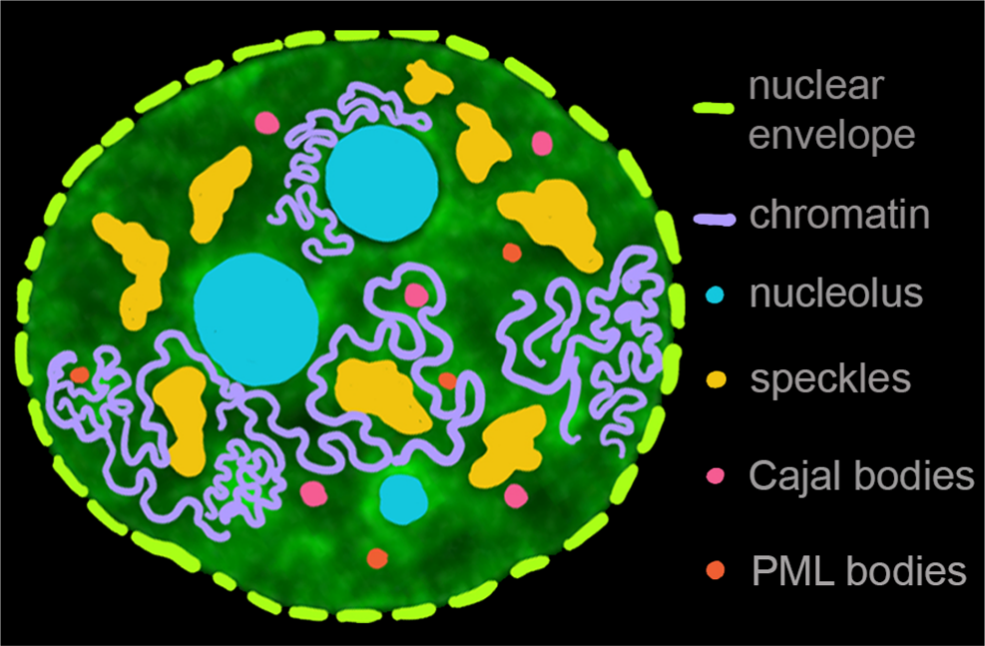
Heterogeneity of the cell nucleus. Cartoon illustrating the following nuclear components: nuclear envelope (*green*), chromatin fiber (*purple*), and subnuclear bodies such as nucleoli (*blue*), speckles (*yellow*), Cajal bodies (*pink*), and PML bodies (*red*), overlayed with a micrograph of a human cell nucleus with fluorescently labeled chromatin (H2B-GFP, *green signal*)

The major component of the nucleus is the chromatin fiber composed of DNA wrapped around protein particles, nucleosomes, made of the histone proteins and resembling a *beads-on-a-string* structure (Alberts et al. 2014). In the human genome, about 2 m of DNA are packed inside a nucleus of roughly 10 μm diameter (Fig. 1) (Alberts et al. 2014). The chromatin fiber is further folded into a 3D conformation, the static structure of which has been elucidated in great detail by chromosome conformation capture techniques (e.g., HiC), which measure probabilities of specific genomic sequences being in physical proximity of each other (Lieberman-Aiden et al. 2009; Dekker et al. 2013; Gibcus and Dekker 2013; Bonev and Cavalli 2016). HiC revealed that chromatin fiber is hierarchically organized with increasing length scale: First, it makes loops, leading to formation of topologically associated domains, which are further assembled into A and B compartments, corresponding to transcriptionally active and inactive compartments, respectively, and finally into chromosome territories (Lieberman-Aiden et al. 2009; Cremer and Cremer 2010; Dekker et al. 2013; Gibcus and Dekker 2013; Bonev and Cavalli 2016). Moreover, HiC identified functional sequence elements, e.g., CTCF, involved in the maintenance of genome folding (Dixon et al. 2012; Nuebler et al. 2018). Optical and electron microscopy revealed that chromatin density distribution inside human nuclei is quite heterogeneous (Ou et al. 2017; Boettiger and Murphy 2020; Boopathi et al. 2020) with major chromatin compartments being euchromatin and heterochromatin (Solovei et al. 2016; Bonev and Cavalli 2016; Van Steensel and Belmont 2017). The former represents loosely packed transcriptionally active chromatin, whereas the latter corresponds to more condensed chromatin that houses predominantly silenced genes. Heterochromatin has been found to localize mainly at the nuclear periphery next to the nuclear lamina, at the nucleolar surface and some in the nuclear interior (Fig. 1, purple) (Solovei et al. 2016; Bonev and Cavalli 2016; Van Steensel and Belmont 2017). An exception to this organization has been observed in retinal cells of nocturnal animals, where heterochromatin is located in the nuclear center and believed to serve as a lens aiding in photon collection (Solovei et al. 2009; Falk et al. 2019).

Embedded in chromatin are subnuclear bodies, structures ranging in size from 50 nm to 3 μm, including nucleoli, speckles, and Cajal and PML bodies, all of which present an excluded volume for the chromatin fiber (Fig. 1) (Mao et al. 2011; Staněk and Fox 2017). Interestingly, these organelles comprised of RNA and proteins are membrane-less, without any physical boundaries separating them from chromatin and nucleoplasm (Mao et al. 2011; Staněk and Fox 2017). The largest subnuclear structure is the nucleolus, a site of ribosomal biogenesis and a critical organelle for cellular cell cycle progression, stress response, and aging (Fig. 1, blue) (Boisvert et al. 2007; Montanaro et al. 2008; Boulon et al. 2010). Nucleoli form at specific genomic loci, termed nucleolar organizer regions, and remain tethered to the rDNA genes for their lifetime (McClintock 1934). Moreover, rDNA transcription is closely linked to nucleolar formation, with nucleoli dissolving upon eliminating this activity (Grob et al. 2014). The nucleolus has been found to behave as a liquid droplet and to form via liquid–liquid phase separation of nucleolar proteins and RNA from the nucleoplasm (Brangwynne et al. 2011; Feric et al. 2016; Caragine et al. 2018). Strikingly, its surface exhibits subtle fluctuations in vivo consistent with a liquid droplet of a very low surface tension (Caragine et al. 2018). Other large structures present in the nucleus and devoid of chromatin are speckles (Fig. 1, yellow). They are responsible for splicing, the post-transcriptional processing of RNA, and were also shown to exhibit liquid-like properties (Marzahn et al. 2016; Kim et al. 2019). Smaller structures like Cajal bodies (Fig. 1, pink) and PML bodies (Fig. 1, red) were found to participate in telomere maintenance and transcriptional regulation, respectively; however, their full functionality remains unknown (Platani et al. 2002; Görisch et al. 2004; Jády et al. 2006).

Chromatin and subnuclear bodies are immersed in the nucleoplasmic fluid, which is aqueous in its nature, enriched with nuclear molecular machinery and its products (Alberts et al. 2014). The nucleoplasmic composition likely varies in space and time with the progress of nuclear processes (Liang et al. 2009; Dross et al. 2009; Erdel et al. 2015). Thus, in first order, the nuclear content can be viewed as a colloidal suspension containing polydisperse colloidal particles embedded in a heterogeneous polymer solution in a multicomponent solvent (Fig. 1). To extract the complex behavior of the system and to understand the biological function and underlying physics of its components, its heterogeneity must be taken into account. The stark degree of heterogeneity of the nuclear content requires detailed approaches focused on specific nuclear components and their behavior as well as mapping of their respective interactions in different local microenvironments across the nucleus.

## Dynamics of nucleus and its constituents

### Nuclear reorganization and shape fluctuations

**Fig. 2:**
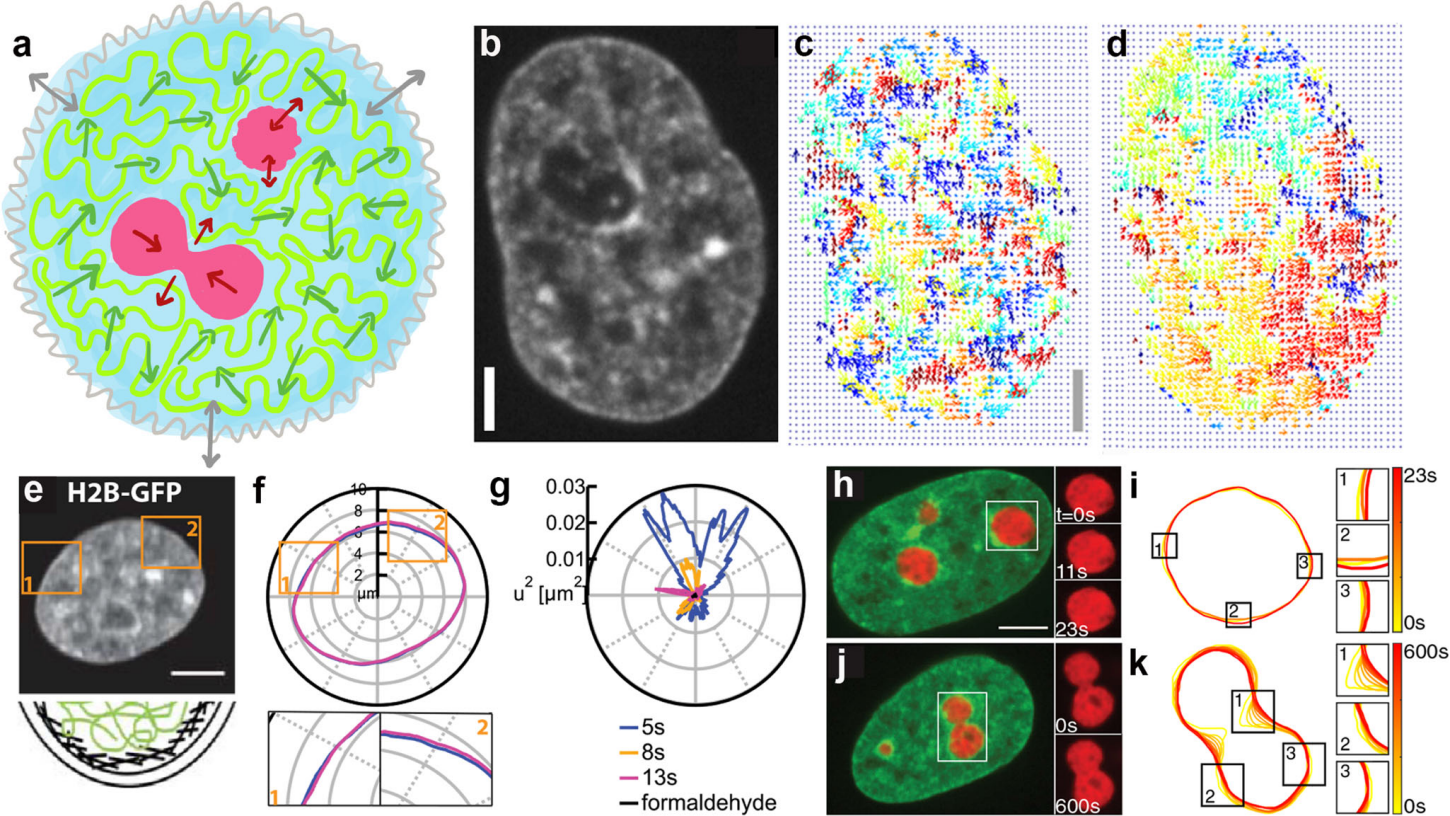
Dynamics of nuclear components. **a** Cartoon illustrating motions of different nuclear components: undulations of the nuclear envelope (*gray*), coalescence and surface fluctuations of nucleoli (*pink*), and chromatin dynamics (*green*), with arrows indicating their respective motions. **b** Micrograph of a human cell nucleus with fluorescently labeled chromatin (H2B-GFP) and maps of chromatin motions by Displacement Correlation Spectroscopy (DCS) obtained at **c** short timescale, Δ*t* = 0.25 s, and **d** long timescale, Δ*t* = 10 s. Scale bar, 2 μm. **b–d** adapted from Zidovska et al. (2013). **e** Micrograph of a human cell nucleus with fluorescently labeled chromatin (H2B-GFP) and a cartoon illustrating the chromatin fiber (*green*) next to the nuclear envelope (*black*). Scale bar, 5 μm. **f** Nuclear contours of the nucleus from (**e**) at different times, with insets showing enlarged view of two areas with visible contour fluctuations. **g** Nuclear shape fluctuations, *u*^2^(*ϕ*,*t*), where *u*(*ϕ*,*t*) is the instantaneous deviation of the contour at polar angle *ϕ* and time *t* from the average contour. **e–g** adapted from Chu et al. (2017). **h, j** Micrographs of human cell nuclei with fluorescently labeled chromatin (H2B-GFP, *green*) and nucleoli (NPM-mApple, *red*), insets show an enlarged view of the boxed nucleoli at different times. Scale bar, 5 μm. **i, k** Contours of nucleoli from (**h, j**) at different times, with insets highlighting nucleolar surface fluctuations (**i**) and shape changes during nucleolar coalescence (**k**). **h–k** adapted from Caragine et al. (2018)

To perform their respective biological functions, the nucleus and its constituents have to be highly dynamic, constantly rearranging and restructuring (Fig. 2a) (Alberts et al. 2014). The nucleus as a whole undergoes a major reorganization during the cell cycle. At the beginning of interphase, the time between two cell divisions, the nuclear envelope forms around mitotic chromosomes decondensing into loosely packed chromosomes, each of which corresponds to a single linear polymer and constitutes a chromosome territory (Cremer and Cremer 2010; Alberts et al. 2014). The genome is then duplicated and later condensed back into mitotic chromosomes facilitating chromosome segregation during the cell division (Alberts et al. 2014). During interphase, the size of the nucleus monotonously increases over hours (Chu et al. 2017), while exhibiting small oscillations of the nuclear area over minutes (Talwar et al. 2013; Makhija et al. 2016) and fast undulations, flickering, of the nuclear envelope over seconds (Chu et al. 2017). The amplitude of the nuclear envelope fluctuations (as depicted in Fig. 2e–g) steadily decreases during the interphase and thus can be utilized as a reliable cell cycle stage indicator in live cells (Chu et al. 2017). The reduction in the nuclear shape fluctuations with progressing cell cycle has been attributed to the increase in the bending rigidity of the nuclear envelope by the gradual deposition of lamin intermediate filaments, although a contribution from cell–cycle–specific forces cannot be ruled out (Chu et al. 2017). Finally, as the cell enters mitosis, the nuclear envelope dissolves, and the nucleus as an entity ceases to exist (Alberts et al. 2014).

### Chromatin dynamics

Numerous site-specific DNA transactions such as transcription, replication, and DNA repair contribute to chromatin dynamics during the cell cycle, giving rise to chromatin dynamics at different timescales and length scales. Over the past two decades, chromatin dynamics has been investigated by tracking motions of fluorescently tagged nuclear proteins visualizing structures of interest such as nucleosomes (Xu et al. 2018; Nagashima et al. 2019; Ashwin et al. 2019), single genes (Marshall et al. 1997; Belmont and Straight 1998; Levi et al. 2005; Chuang et al. 2006; Bronstein et al. 2009; Weber et al. 2012; Chen et al. 2013; Lampo et al. 2016; Germier et al. 2017; Amitai and Holcman 2018; Khanna et al. 2019; Vivante et al. 2020), nuclear proteins, enzymes and machineries (Misteli 2001; Carmo-Fonseca et al. 2002; Darzacq et al. 2007; Stixová et al. 2011; Cisse et al. 2013; Hinde et al. 2014; Eaton and Zidovska 2020), and subchromosomal foci (Bornfleth et al. 1999; Albiez et al. 2006), as well as entire chromosome territories (Zink et al. 1998; Edelmann et al. 2001). Furthermore, experiments measuring fluorescence recovery after photobleaching (Abney et al. 1997; Misteli et al. 2000; Phair and Misteli 2000; Kimura and Cook 2001) and photoactivation (Mora-Bermúdez et al. 2007; Wiesmeijer et al. 2008) of nuclear proteins have revealed their peculiar kinetics. All of these approaches have contributed to our understanding of dynamic processes in the cell nucleus. Chromatin dynamics was shown to be mostly subdiffusive to diffusive with occasional directed motion. While single particle tracking approaches are highly informative (Shukron et al. 2019), reporting about the local chromatin dynamics of a tracked entity, it is unclear how these local motions relate to each other on a larger, genome-wide scale.

To elucidate the large-scale genome-wide chromatin motions in vivo, a new spectroscopy-based method Displacement Correlation Spectroscopy (DCS) was recently developed (Zidovska et al. 2013). DCS is a microscopy-based image correlation method, which introduced spatiotemporal spectroscopy analysis into the dynamic image correlation processing. It maps chromatin dynamics over time intervals as shown in Fig. 2b–d, while concurrently sampling all time intervals accessible by the experiment (Zidovska et al. 2013). Using transgenic histones H2B-GFP as markers of chromatin position and high-resolution spinning disc confocal microscopy (Fig. 2b), this noninvasive technique enables measurement of chromatin dynamics in real time across the entire nucleus in live cells, while simultaneously probing different timescales and length scales (Fig. 2c–d). Using DCS, chromatin dynamics was found to be subdiffusive with two distinct time and length scales: (i) fast, local motion, and (ii) slower, coherent motion (Zidovska et al. 2013). While the first had been observed before by single particle tracking, the slower, correlated motion is new and has major implications for the organization of nuclei on micron-second scales (Zidovska 2020). Importantly, these motions happen in the nucleus concurrently and superposed. Domains of coherent motion ($\sim $ 3–5μm) reach across chromosome territories, suggesting some types of coupling of motion over scales that are huge compared to individual genes (Zidovska et al. 2013). The discovery of coherent chromatin motion was later corroborated by high-resolution imaging of the local motion of single nucleosomes and replication domains and DCS-like spectroscopy analysis in U2OS cells (Nozaki et al. 2017; Xiang et al. 2018; Shaban et al. 2018). While the biological role of coherent motion is yet to be uncovered, it leads to physical motion of the entire genome, thus likely impacting gene regulation via local changes in rates and molecular transport in the nucleus. It may account for apparently directed movements of tagged genes that have been reported in the literature and whose mechanism is unknown (Marshall et al. 1997; Levi et al. 2005; Chuang et al. 2006). These large-scale coupled motions were ATP-dependent and independent of the cytoplasmic cytoskeleton (Zidovska et al. 2013). Perturbation of major nuclear ATPases such as DNA polymerase, RNA polymerase II, and topoisomerase II caused local displacements to increase, but eliminated coherence, i.e., local motions became uncoupled (Zidovska et al. 2013; Shaban et al. 2018). These observations revealed coherent motions to be an emergent property of the active chromatin dynamics, suggesting that gene-level activity might lead to the nucleus-wide motions (Zidovska 2020).

Motivated by the DCS observations, theoretical approaches were developed to further explore the role of activity in chromatin dynamics. First, a hydrodynamic theory accounting for active chromatin dynamics within the nucleoplasmic fluid was developed (Bruinsma et al. 2014). This theory introduces two types of active events that can act on the chromatin fiber: scalar events and vector events. Scalar events correspond to local condensation and decondensation of the chromatin fiber, which can be caused for example by chromatin remodelers (Bruinsma et al. 2014; Racki and Narlikar 2008). Such events do not have a direction, only a magnitude. In contrast, vector events represent activity induced by nuclear enzymes such as RNA polymerase II, helicase, and topoisimerase II, which can be described by a force dipole. A force dipole consists of two equally large but opposing forces, thus possessing both magnitude and a direction. It corresponds to the force that the enzyme exerts on the chromatin fiber and the opposing force applied on the surrounding fluid due to Newton’s 3rd law. Thus, the presence of force dipoles leads to local nucleoplasmic flows, which in turn interact with the chromatin fiber. Moreover, this theory predicts that vector events can lead to large-scale fluctuations due to dipolar interactions, suggesting that collective alignment of force dipoles can lead to large-scale coherence of chromatin dynamics, whereas the scalar events give rise to chromatin concentration fluctuations at short length scales (Bruinsma et al. 2014).

The effect of force dipoles on chromatin dynamics within the nucleoplasm was further investigated by computational simulations, which revealed that extensile (outward) dipolar forces can give rise to the chromatin coherent motion as well as large-scale nucleoplasmic flows (Saintillan et al. 2018). In contrast, contractile (inwards) dipolar forces led to a seemingly accelerated Brownian dynamics (Saintillan et al. 2018). Nucleoplasmic flows due to chromatin activity may likely contribute to the transport of molecular machinery within the nucleus, which would otherwise be diffusion-limited (Saintillan et al. 2018). Interestingly, hydrodynamic-free approaches accounting for chromatin activity were also able to reproduce the large-scale chromatin coherence observed by DCS (Liu et al. 2018; Shi et al. 2018; Di Pierro et al. 2018). In these models, chromatin fiber conformation is given by a quasi-equilibrium energy landscape or informed by HiC experiments and activity applied either implicitly via effective temperature in a quasi-equilibrium (Di Pierro et al. 2018) or explicitly via an isotropic noise (Liu et al. 2018). In addition, chromatin dynamics was found to resemble glassy behavior with many different types of subdiffusive motion (Shi et al. 2018). Strikingly, while hydrodynamic models suggest a key role of the nucleoplasm in chromatin coherent motion, it is possible that in the hydrodynamic-free models the nucleoplasm may be involved in achieving the preferred chromatin fiber conformations used in those models. Lastly, it is important to note that the nucleoplasm itself may need to be considered an active fluid, as it contains a wealth of nuclear enzymes and subnuclear bodies, whose potential activity could contribute to active flows (Zidovska 2020).

### Dynamics of subnuclear bodies

Within the dynamic chromatin network, there are subnuclear bodies such as nucleoli, speckles, and Cajal bodies, which can undergo their own dynamical events. These are membrane-less structures exhibiting liquid-like behavior. The archetype of these liquid condensates is the nucleolus (Fig. 2h, red), which was found quite dynamic in vivo (Brangwynne et al. 2011; Weber and Brangwynne 2015; Caragine et al. 2018, 2019). First, nucleolar formation is nucleated at specific genomic sites (nucleolar organizer regions, NORs), which encode for ribosomal genes (rDNA) (McClintock 1934; Ritossa and Spiegelman 1965; Wallace and Birnstiel 1966). Nucleoli then form via the liquid–liquid phase separation of nucleolar proteins from the nucleoplasm (Brangwynne et al. 2011; Berry et al. 2015; Feric et al. 2016). In addition, active recruitment of participating proteins may also play a role (Falahati and Wieschaus 2017). Nucleoli remain attached to rDNA for their lifetime; therefore, the number of nucleoli in the nucleus is limited by the number of NORs in the genome (Amenta 1961; Sullivan et al. 2001). Although tethered to chromatin permanently in somatic cells, nucleoli exhibit translatory motion, albeit impeded by this attachment (Caragine et al. 2019). The nucleolar number then decreases during the cell cycle via fusion of smaller nucleoli into larger ones (Caragine et al. 2018, 2019). Interestingly, members of nucleolar pairs were shown to undergo correlated motion if they were in approach to fuse, while otherwise they exhibited independent motions (Caragine et al. 2019). A careful inspection of the kinetics of nucleolar fusion as shown in Fig. 2j–k revealed them to be consistent with coalescence of liquid droplets in a surrounding fluid of higher viscosity. Moreover, human nucleoli were shown to exhibit subtle, yet measurable surface fluctuations depicted in Fig. 2h–i, consistent with liquid droplets of very low surface tension (Caragine et al. 2018). The nucleolar interface was found to be actively maintained by ATP-dependent processes related to chromatin packing and transcription (Caragine et al. 2019).

Similarly, other types of liquid condensates in the nucleus such as speckles (Kim et al. 2019) and Cajal bodies (Platani et al. 2002; Görisch et al. 2004; Jády et al. 2006; Schmidt et al. 2016) were also found to move inside the nucleus. Upon transcriptional inhibition, speckles were found to change their shape on timescales of minutes, move towards each other in a directionally correlated way, and coalesce like liquid droplets (Kim et al. 2019). Cajal bodies exhibit ATP-dependent subdiffusive motion with an intermittent association towards the surrounding chromatin (Platani et al. 2002). Strikingly, dynamics of nuclear bodies is tightly coupled to active processes in the nucleus, emphasizing the non-equilibrium nature of their physical behavior.

## Nuclear compartmentalization via phase separations

**Fig. 3:**
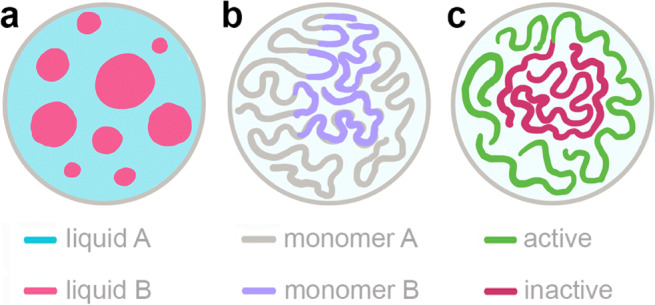
Nuclear compartmentalization via phase separations. Schematics illustrating different types of phase separations implicated in the nuclear organization: **a** liquid-liquid phase separation of liquid components (liquid A, *blue*, and liquid B, *pink*), **b** microphase separation of chromatin fiber as a block-copolymer (monomer A, *gray*, and monomer B, *purple*), and **c** activity-driven phase separation due to local active processes (active, *green*, and inactive, *red*)

The nucleoplasm presents a solvent to polymers such as the chromatin fiber and RNA, as well as to colloidal particles in the form of liquid condensates and protein aggregates in the nucleus. Thus, it is directly involved in the fluid-mediated interactions among the respective nuclear components. Moreover, the nucleoplasm contributes to both nuclear heterogeneity as well as dynamics by carrying the molecular machinery needed for nuclear processes as well as their products. Inevitably, its composition must vary in space and time in vivo. In fact, numerous nucleoplasmic proteins were shown to phase separate from the nucleoplasm and form liquid condensates via liquid–liquid phase separation (LLPS) as illustrated in Fig. 3a. In addition to nucleoli and speckles, which we discussed earlier, transcription machinery located at active genes (Cho et al. 2018; Guo et al. 2019), DNA-repair machinery at double-stranded DNA breaks (Kilic et al. 2019; Pessina et al. 2019) and HP1 proteins at heterochromatin (Strom et al. 2017; Larson et al. 2017) were all shown to form liquid-like condensates. Thus, LLPS of various nucleoplasmic components can provide nuclear compartments generating local chemical reactors dedicated to specific biological functions, e.g., ribosome biogenesis or heterochromatin formation (Fig. 3a).

Presently, it remains an open question how to build an integrated physical picture including concurrent LLPS of multiple components into their distinct functional compartments with the nucleoplasm as the surrounding liquid supplying all necessary components. Moreover, other phase-separation driven processes might compete or complement each other with nucleoplasmic LLPS. For example, while LLPS of heterochromatin HP1 proteins was shown to drive the formation of heterochromatin in genome (Strom et al. 2017; Larson et al. 2017), computational simulations suggested that HP1 association with parts of the chromatin fiber could lead to microphase polymer separation of heterochromatin and euchromatin in the absence of a solvent and thus without any hydrodynamic interactions (MacPherson et al. 2018; Falk et al. 2019). In these studies, chromatin fiber was considered to be a block-copolymer with different types of monomers comprising different polymer blocks as well as varying interactions between distinct monomer types, e.g., attraction of HP1-bound monomers (Fig. 3b). To add to this complexity, chromatin fiber itself was shown to be able to undergo local LLPS in the nucleoplasm (Gibson et al. 2019).

Furthermore, it has to be noted that all nuclear constituents (chromatin, subnuclear bodies, and nucleoplasm) are non-equilibrium systems containing both active (i.e., ATP-driven) and passive (i.e., thermally driven) components. Strikingly, both colloidal and polymer mixtures comprised of active and passive components were shown to phase separate their active and passive entities (Stenhammar et al. 2015; Smrek and Kremer 2017). In fact, in the case of polymers, such phase separation was proposed to play a role in the formation of euchromatin and heterochromatin, the respective transcriptionally active and inactive parts of the genome (Ganai et al. 2014; Smrek and Kremer 2017; Shi et al. 2018). Hence, the presence of the activity must also be considered when interrogating the nucleus and its organization and dynamics (Fig. 3c). In the light of these observations, it is conceivable that the combined effects of microphase, activity-driven and liquid–liquid phase separations might need to be considered. Moreover, interactions among these effects may lead to new physical phenomena.

## Emergent nuclear rheology in vivo

Given the heterogeneous, dynamic, and non-equilibrium nature of the nucleus, its material properties are inevitably highly complex. Nuclear heterogeneity highlights the composite character of the nucleus, while the non-equilibrium dynamics leads to an overall emergent behavior, part of which is its rheology. Elucidating material properties of the nucleus as a whole as well as of its components is crucial for revealing the biophysical origins of its underlying physiology and building a mechanistic picture of the nucleus.

**Fig. 4:**
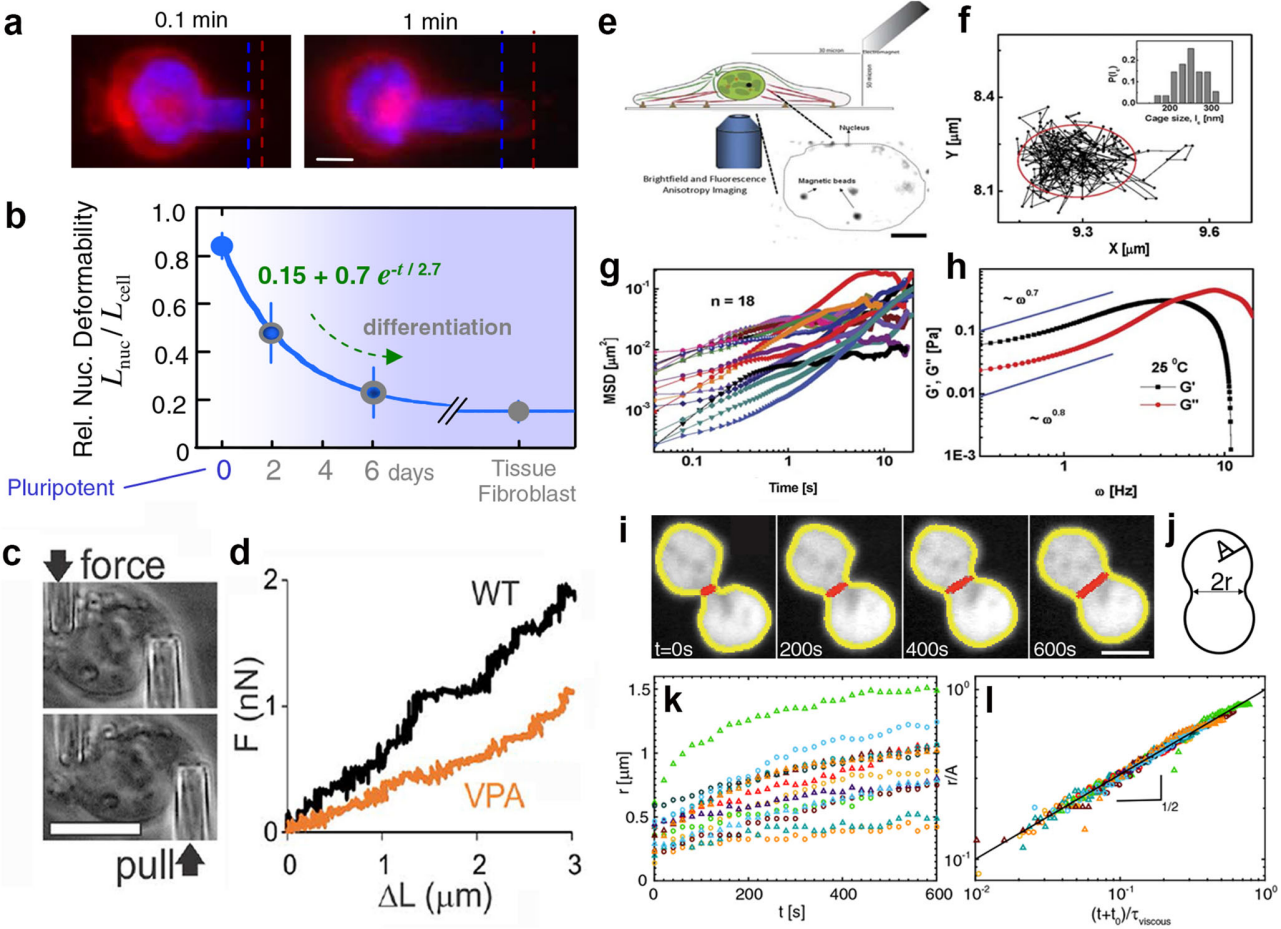
Experimental techniques for nuclear rheology in vivo. **a** Micropipette aspiration of human stem cell with fluorescently labeled nucleus (*blue*) and plasma membrane (*red*). Scale bar, 3 μm. **b** Ratio of nuclear to cytoplasmic extension, *L*_*nuc*_/*L*_*cell*_, changes during cell differentiation, with the nucleus stiffening relative to cytoplasm. **a, b** adapted from Pajerowski et al. (2007). **c** Micromanipulation force measurement of an isolated nucleus observing nuclear extension upon movement of the *pull* pipette while measuring force through deflection of the *force* pipette. Scale bar, 10 μm. **d** Force-extension plot for nuclei treated with valproic acid (VPA) and untreated nuclei (WT). **c, d** adapted from Stephens et al. (2018). **e** Single particle tracking analysis of particles in the nucleus. Schematics of the experimental setup, with an electromagnet used to apply forces on paramagnetic particles injected into the nucleus. Inset shows a brightfield image of particles inside the nucleus, outlined by the dotted line. Scale bar, 5 μm. **f** Trajectory of a bead in the nucleus of a living cell with inset showing histogram of cage sizes measured over 30 beads. **g** Mean square displacement (MSD) as a function of time for beads in the nuclei in the absence of force. **h** Shear and loss moduli, $G^{\prime }$ and $G^{\prime \prime }$, as a function of frequency *ω*. **e–h** adapted from Hameed et al. (2012). **i** Nucleolar coalescence serves as a rheological probe of the nucleus by analyzing the nucleolar shape (NPM-mApple, *white signal*) in live cells in over time. The nucleolar contour (*yellow line*) and neck connecting two coalescing nucleoli (*red line*) are determined at each time point. Scale bar, 2 μm. **j** Cartoon illustrating measured variables: neck diameter, 2*r*, and average radius of the two nucleoli before fusion, *A*. **k** Neck radius *r* as a function of time for 14 fusion events. **l** Rescaled neck radius *r*/*A* as a function of rescaled time. Solid line represents *t*^1/2^. **i–l** adapted from Caragine et al. (2018)

The bulk rheology of the entire nucleus has been probed using micropipette aspiration (Fig. 4a–b) (Dahl et al. 2004, 2005; Pajerowski et al. 2007) and micromanipulation techniques (Fig. 4c–d) (Stephens et al. 2017; Stephens et al. 2018), showing a complex viscoelastic behavior of the composite nucleus consistent with multiple relaxation processes. Moreover, combined with biochemical and transgenic alterations of nuclear components, these techniques revealed mechanical contributions of the nuclear envelope and chromatin (Dahl et al. 2004, 2005; Pajerowski et al. 2007; Stephens et al. 2017; Stephens et al. 2018). For example, micropippette aspiration methods in live human stem cells revealed the predominantly elastic contribution of the nuclear envelope and chromatin being more viscous (Pajerowski et al. 2007), while the micromanipulation techniques revealed that chromatin governs resistance to small nuclear deformations in isolated nuclei (Stephens et al. 2017). These results further demonstrate the criticality of illuminating mechanical contributions of individual nuclear components.

To explore the rheology of the nuclear interior, passive and active microrheology approaches were employed. The former relies on the injection of nonmagnetic particles inside the cell nucleus and follows their displacement in time, while the latter uses magnetic particles and measures their displacement in response to application of a known external magnetic force (Fig. 4e). In both cases, the displacement of the particle informs on the mechanical response of the media surrounding the particle (Fig. 4f–h). Microrheology approaches found the viscosity of the nucleoplasm to be 10 − 10^3^ Pas and elastic modulus of 10^− 1^ − 10^3^ Pa (Tseng et al. 2004; de Vries et al. 2007; Celedon et al. 2011; Hameed et al. 2012). These results range over several orders of magnitude, possibly due to the heterogeneity of the nucleus as well as differences in probe size. Particles of different sizes are sensitive to different features of the system they are embedded in, and therefore report on the rheology at different length scales. The heterogeneity of the nucleus, which we discussed earlier, suggests that a position of the rheological probe will also impact its readout, reporting on different local microenvironments. Recently, a new way of introducing artificial particles into the nucleus was developed, using synthetic droplets whose molecular components are expressed by transgenically modified cells (Shin et al. 2018). These components then assemble upon a light stimulus into droplets, which can be used as rheological probes (Shin et al. 2018).

In addition, a noninvasive microrheology method was recently developed that uses intrinsic dynamics of naturally occurring nuclear structures to probe the nuclear rheology (Caragine et al. 2018). This approach uses spontaneous physiological dynamics of nuclear components such as the nucleolus to probe the material properties of the nucleus and its constituents. Specifically, two types of nucleolar motions were employed: its surface fluctuations and fusion events. The nucleolar surface fluctuations report on the surface tension of the nucleolus-nucleoplasm interface (Fig. 2h–i). Analysis of these fluctuations in vivo revealed a surface tension of $\sim 10^{-6}$ N*m*^− 1^ (Caragine et al. 2018), a surface tension $\sim 10^{4}$ times lower than that of a water droplet in air. Such low surface tensions have been measured for interfaces in polymer-colloidal mixtures (Aarts et al. 2004) and frog oocyte nucleoli (Brangwynne et al. 2011; Feric et al. 2016). Furthermore, a careful observation of the kinetics of the nucleolar coalescence revealed which forces dominate the process (Fig. 2j–k). Specifically, this is reflected by the growth kinetics of the neck connecting two coalescing nucleoli as shown in Fig. 4i–l (Paulsen et al. 2014; Caragine et al. 2018). In the case of nucleoli, the viscous forces caused by the external fluid were found to oppose the capillary forces driving the coalescence, revealing a viscosity of the surrounding nucleoplasmic fluid to be $\sim 10^{3}$ Pas (Caragine et al. 2018).

It has to be noted that in all these techniques above, the materials tested were assumed to be in thermodynamic equilibrium. However, as we discussed earlier, the nucleus is far from equilibrium. Thus, it is important that we treat the material properties obtained as effective or apparent properties that the active system appears to have if it were in equilibrium. Hence, the rheology observed is truly an emergent rheology. The case in point is the nucleolar surface, which upon ATP-depletion loses its smoothness, visibly decreasing its surface tension (Caragine et al. 2019). Similarly, the shape of liquid-like speckles dramatically alters its aspect ratio upon transcriptional inhibition (Kim et al. 2019). Thus, the non-equilibrium material properties are a result of participating active forces leading to different properties as would be measured for passive materials.

In light of techniques such as microrheology, methods for measuring dynamics inside the cell nucleus—including those reviewed here in an earlier section—can inform on material properties of the nucleus and its components. However, inferring rheological properties from dynamics is a challenging problem. Indeed, such inference must take into account spatial heterogeneity and intrinsic activity, but theoretical frameworks for such non-equilibrium systems are currently missing. The rich phenomenology of the cell nucleus may guide new non-equilibrium treatments.

## Conclusions and perspective

In summary, the cell nucleus is a heterogeneous, multicomponent system, functionally compartmentalized via phase separations and far from thermodynamic equilibrium. It contains many molecular components participating in active energy-dissipating processes, leading to new effects and phenomenology. Material properties of nuclear components impact the timescales and length scales of nuclear process, most prominently those of the DNA-related biochemical transactions, which constitute an integral part of the central dogma of molecular biology. Hence, emergent rheology of the cell nucleus effectively impacts all cellular processes.

Remarkably, the nucleus is compartmentalized into functional regions separating phases of different physical, material, and chemical properties. These phases serve as sites of active processes carrying out specific biological functions, such as polymerases and transcription factors forming a functional liquid condensates or DNA repair machinery phase separating at DNA double-stranded breaks. Thus, we can view the nucleus as being actively patterned via phase separations and activity-generating local reactors, which in turn contribute to the patterning itself. There is still a long way to go to decouple all of the effects and biological processes as they occur concurrently and superposed in the cell nucleus. Future in vivo studies are needed to investigate the biophysical origins of the complex phenomenology of physiological behavior of the nucleus. In addition, in vitro studies might recapitulate its key features and examine its underlying mechanisms. Moreover, simultaneous orthogonal biochemical and biophysical processes may need to be explored within their common multicomponent phase diagram both in vivo and in vitro. Furthermore, the collective phenomena that occur in this active system may strongly contribute to such emergent behavior.

Similar to other living systems, the nucleus presents an intricate interplay of heterogeneity and non-equili-brium activity posing new challenges for biologists and physicists alike. It calls for new experimental and analytical approaches rooted in soft condensed matter physics, biophysics, and statistical mechanics while connecting to the biochemistry and molecular biology of the nucleus. Moreover, phenomena found in this system may teach us new non-equilibrium physics. Such knowledge is critical also from biomedical perspective identifying potential physical parameters as readouts for diagnostic tools and therapy design for diseases rooted in malfunctions of nuclear constituents.

## The Michèle Auger Award for Young Scientists’ Independent Research

In late 2018, long time Editorial Board Member of Biophysical Reviews journal, Professor Michèle Auger, sadly succumbed to illness. As a mark of our respect for Michèle, the Biophysical Reviews’ Editorial Board, together with the kind support of Springer-Nature Corporation, created a perpetual memorial award in honor of her life and service. The, ‘Michèle Auger Award for Young Scientists’ Independent Research’, is to be granted each year to a single candidate performing biophysical research, who at the time of application is under 40 years of age. The award consists of a plaque and a free personal subscription to the journal along with an invitation to submit a single author review article to Biophysical Reviews.

## Michèle Auger (1963 - 2018)

Born in GrandMère, Quebec and raised in TroisRivières, Michèle Auger enrolled first in biophysics at the Université du Québec à Trois-Rivières, only to later transfer to chemistry. After obtaining her B. Sc. in 1985, Michèle joined the group of Prof. Ian C.P. Smith at the University of Ottawa/National Research Council of Canada to pursue her Ph. D. studies in biophysics. After graduating from the University of Ottawa in 1990, Michèle refined her skills in solid state NMR as a postdoctoral fellow in the group of Prof. Robert G. Griffin at the Massachusetts Institute of Technology. Michèle joined the Department of Chemistry at the University of Laval in 1991 as an Assistant Professor and recipient of an NSERC Women’s Faculty Award. She was promoted to Associate Professor in 1996 and then Professor in 2000 where she remained until 2018. Michèle’s research involved using solid state NMR to study (i) the interaction of proteins, peptides and drugs with phospholipid membranes, and (ii) biopolymers such as spider silk. Michèle served internationally on the Council of the International Union of Pure and Applied Biophysics from 2011 - 2017 and was an Editorial Board Member of Biophysical Reviews journal from 2011-2018. Brilliant, creative, dedicated to the scientific and academic communities, Michèle displayed admirable professional, ethical and leadership qualities. She is remembered as a dedicated, generous, and inspirational scientist who touched the lives of many through her friendship, teaching and kindness. (This short foreword is adapted from a longer memorial published on the IUPAB Newsletter #70 http://iupab.org/wp-content/uploads/2019/02/IUPAB-news-70-2-1.pdf)

## Alexandra Zidovska: Recipient of the Michèle Auger Award for Young Scientists’ Independent Research 2020

Prof. Alexandra Zidovska is an experimental physicist studying physical phenomena in biological systems and materials. Her career-long passion for biophysics has its roots in her undergraduate research work under Prof. Erich Sackmann at the Technical University Munich in Germany, where she earned her Diplom or Master’s Degree in Physics in 2003. She used reflection interference contrast microscopy (RICM) and magnetic tweezers to study micromechanical properties of the cell membrane and filopodia of macrophages. Her undergraduate work revealed that the cell membrane of macrophages exhibits undulations that impede their adhesion, which may be critical for their ability to fight pathogens in human body. For her PhD studies, Alexandra moved on to the University of California, Santa Barbara where she worked in the lab of Prof. Cyrus Safinya in the Materials Department and graduated in 2008. She credits Prof. Safinya with instilling in her a sense of taste in problems and how to bring the right tools to the study of these problems. Her doctoral work focused on studies of the structure-function relationship of lipid-DNA self-assemblies used for gene delivery. She investigated liquid crystalline structures of lipid-DNA self-assemblies and phase behavior of exotic highly charged dendrimer lipids using small angle X-ray scattering (SAXS), confocal microscopy and cryogenic transmission electron microscopy (cryoTEM). Her graduate work produced an impressive record of papers in esteemed journals, including the discovery of a new phase of liposomes, the block liposomes.

Alexandra then conducted her post-doctoral research at Harvard University, where she worked jointly in the labs of Prof. Timothy Mitchison at Harvard Medical School and Prof. David Weitz in the Physics Department. Seeking yet greater challenges in her research, she made a brave change of direction and began a quest to understand the physics of the cell nucleus and the genome. It was during this time that she developed a new method Displacement Correlation Spectroscopy (DCS) for mapping and quantifying chromatin motions simultaneously across the entire cell nucleus. DCS employs high-resolution spinning disc confocal microscopy and measures chromatin displacements by image correlation techniques. Because the method reveals the nucleus-wide motions maps across the entire temporal spectrum, the method has proven to be a major leap forward for the field. Indeed, Alexandra’s work revealed a new form of motion of the genome in which large regions of chromatin move together coherently over extended periods of time. Her work earned her the prestigious Damon Runyon Cancer Research Fellowship among other honors.

Since 2014, Alexandra’s work as Assistant Professor of Physics and research group leader at New York University’s Center for Soft Matter Research has continued to uncover the physics and inner workings of the cell nucleus. She leveraged early-career awards such as The Pathway to Independence Award from the National Institutes of Health and the CAREER Award from the National Science Foundation, as well as a New York University Whitehead Fellowship for Junior Faculty in Biomedical and Biological Sciences, to establish a world-class experimental lab that in its first years can already proudly claim several major discoveries. One line of work follows up on her discovery of coherent chromatin motion, showing that its origin lies in active processes such as transcription occurring at the single-gene level and whose influence is amplified genome-wide due to hydrodynamic self-interactions of the chromatin fiber. She and her team have also shown that the nuclear envelope exhibits persistent flickering motions that had gone unnoticed previously and whose origin also lies in active cellular process. Very recently, the Zidovska lab has shown how the intrinsic motions and coalescence of subnuclear bodies such as nucleoli can be exploited to infer material properties of the nuclear environment. Some of these exciting results are reviewed in her accompanying article, which is a stirring and moving account of the cell nucleus that seems to be in perpetual motion.

In an example of life imitating science, the themes of motion, flow, activity, energy and purpose permeate Alexandra’s pursuits outside of the lab. She is a lifelong swimmer who uses her pool time to stoke her competitive flame, balanced by a healthy dose of flow yoga. She also grounds herself through creative outlets such as painting and cooking. Alexandra is also passionately engaged in causes related to diversity and inclusion in physics and related sciences. Her lab has an impressive record of recruiting, training and promoting women scientists across all levels, and Alexandra is founder and faculty leader of a new group, NYU WiPhy, dedicated to providing a more welcoming and stimulating environment for women and those from other underrepresented groups in physics.
